# Uncovering Potential Neutrophil-Related Biomarkers for Early AMI Diagnosis

**DOI:** 10.3390/biology15100781

**Published:** 2026-05-14

**Authors:** Yuwei Liu, Yun Zhang, Lucheng Wang, Diru Yao, Ebenezeri Erasto Ngowi, Moussa Omorou, Ning Hou, Weibo Dai, Longlong Wang, Guihua Yue, Aijun Qiao

**Affiliations:** 1 School of Chinese Materia Medica, Nanjing University of Chinese Medicine, Nanjing 210023, China; liuyuwei1075@zidd.ac.cn; 2 State Key Laboratory of Discovery and Utilization of Functional Components in Traditional Chinese Medicine, School of Pharmaceutical Sciences, Guizhou Medical University, Guiyang 550004, China; zhangyun1072@zidd.ac.cn; 3 School of Pharmaceutical Sciences, Guangzhou Medical University, Guangzhou 511436, China; wanglucheng965@zidd.ac.cn (L.W.); houning@gzhmu.edu.cn (N.H.); 4 Zhongshan Institute for Drug Discovery, Shanghai Institute of Materia Medica, Chinese Academy of Sciences, Zhongshan 528400, China; yaodiru0648@zidd.ac.cn (D.Y.); ebenezerierastongowi@simm.ac.cn (E.E.N.); moussa.omorou@mails.ucas.ac.cn (M.O.); 5 Shanghai Institute of Materia Medica, Chinese Academy of Sciences, Shanghai 201203, China; 6 Pharmacology Laboratory, Zhongshan Hospital of Traditional Chinese Medicine, Affiliated with Guangzhou University of Traditional Chinese Medicine, Zhongshan 528400, China; daiweibo007@163.com; 7 Department of Clinical Medicine of Integrated Traditional Chinese and Western Medicine, The Affiliated International Zhuang Medicine Hospital of Guangxi University of Chinese Medicine, Nanning 530200, China; wangll@gxtcmu.edu.cn

**Keywords:** acute myocardial infarction, inflammation, neutrophils, biomarker, machine learning

## Abstract

Early diagnosis of AMI is crucial for improving patient outcomes, yet current clinical tools often lack the requisite sensitivity and specificity for reliable early detection. As neutrophils are the first innate immune responders mobilized following infarction, we employed an integrated multi-omics and machine learning approach to identify neutrophil-driven molecular signatures with diagnostic potential. By analyzing multiple peripheral blood transcriptomic datasets, we conducted differential expression and immune infiltration analyses, followed by machine learning-based feature selection to pinpoint key genes linked to neutrophil activity. Integration of these findings with single-cell transcriptomic data further clarified the neutrophil-specific expression patterns of candidate genes during AMI progression. Using a joint diagnostic model, we identified MCEMP1, NFE2, and AQP9 as the most informative predictors, with MCEMP1 emerging as the primary contributor. Experimental validation in a murine model of myocardial infarction (MI) confirmed rapid upregulation of MCEMP1 after injury, closely mirroring the kinetics of neutrophil infiltration. Collectively, these findings delineate a neutrophil-associated molecular profile of early AMI and highlight MCEMP1 as a promising noninvasive biomarker and a potential therapeutic target for modulating neutrophil-driven myocardial injury.

## Simple Summary

Neutrophils are the first-line innate immune responders to acute myocardial infarction (AMI), positioning their molecular features as promising early diagnostic biomarkers. However, their diagnostic potential in AMI remains largely unexplored. By integrating machine learning-based feature selection and single-cell transcriptomics, we identified key genes associated with neutrophil infiltration during AMI. Among them, mast cell-expressed membrane protein 1 (MCEMP1), nuclear factor, erythroid 2 (NFE2), and aquaporin 9 (AQP9) exhibited strong associations in the early phase of the disease, with MCEMP1 emerging as the most significant predictor in a combined diagnostic model. The expression pattern of MCEMP1 was further confirmed in a myocardial-infarcted (MI) mouse model. These findings reveal early neutrophil-associated molecular signatures in AMI, underscore the potential of MCEMP1 as a circulating biomarker, and offer a new strategy for early clinical diagnosis.

## 1. Introduction

AMI, a predominant subtype of cardiovascular disease, remains a major global health challenge due to its high incidence and mortality rates [1]. The primary pathophysiological mechanism of AMI involves acute thrombosis triggered by the rupture of unstable coronary atherosclerotic (AS) plaques, culminating in severe coronary artery stenosis and irreversible acute ischemic myocardial necrosis [2,3]. The well-documented positive correlation between the duration of myocardial ischemia and patient mortality underscores the critical importance of early and accurate diagnosis to facilitate timely reperfusion or interventional therapy [4]. Conventional diagnostic strategies for AMI primarily rely on electrocardiography (ECG) and the measurement of circulating cardiac troponin T (cTnT) levels [5]. Notably, elevated cTnT levels, for instance, may also be observed in patients with conditions such as heart failure, chronic kidney disease, or myocarditis [6], while individuals with ultimately confirmed AMI can present with a normal ECG [7]. This clinical complexity thus necessitates complementary biomarkers beyond conventional approaches to broaden diagnostic options and enhance accuracy.

The hallmark of AMI is myocardial necrosis accompanied by a robust inflammatory response [8]. As the first immune cells to infiltrate the ischemic myocardium, neutrophils play a central role in orchestrating both the initiation of inflammatory injury and subsequent tissue repair [9]. During the early phase of AMI, neutrophils predominantly exhibit a pro-inflammatory N1 phenotype, exacerbating cardiomyocyte and endothelial damage through the release of reactive oxygen species (ROS), pro-inflammatory mediators, and neutrophil extracellular traps (NETs) [10,11,12]. In the later resolution phase, they transition to an anti-inflammatory N2 phenotype, thereby promoting myocardial healing [12]. This functional duality highlights the complex, time-dependent role of neutrophils in AMI and positions activation-specific biomarkers as promising tools for enhancing early diagnostic precision and prognostic stratification [13].

Here, we conducted immune infiltration analysis on two public transcriptomic datasets to confirm neutrophil enrichment in the peripheral blood of AMI patients. By integrating multiple machine learning algorithms with correlation analysis, we identified five candidate genes (MCEMP1, NFE2, AQP9, suppressor of cytokine signaling 3 (SOCS3), and adrenomedullin (ADM)) associated with neutrophil activation. Analysis of a mouse single-cell transcriptomics dataset (GSE163129) further validated the specific overexpression of MCEMP1, NFE2, and AQP9 in neutrophils from MI models. Furthermore, a combined diagnostic model incorporating these three genes outperformed single-marker performance, with MCEMP1 emerging as the primary contributor and demonstrating considerable potential as a diagnostic biomarker. Finally, the dynamic expression patterns of these candidate genes were confirmed in a murine model of MI.

## 2. Materials and Methods

### 2.1. Data Acquisition and Single-Cell Resolution Validation

To identify and validate key molecular signatures in AMI, transcriptomic profiles were analyzed from three independent datasets available in the National Center for Biotechnology Information Gene Expression Omnibus (GEO): GSE59867 [14], GSE60993 [15], and GSE62646 [16], https://www.ncbi.nlm.nih.gov/geo/ (accessed on 10 October 2025). Only samples collected on the first day of hospital admission were used in this study. These cohorts comprise peripheral blood samples from patients with ST-elevation myocardial infarction (STEMI) and control individuals. Among them, the GSE59867 dataset includes bulk transcriptomic data from 111 STEMI patients and 46 control subjects diagnosed with stable coronary artery disease (CAD) with no prior history of MI. GSE60993 includes peripheral blood profiles from 7 STEMI patients and 7 healthy controls, while GSE62646 comprises samples from 28 STEMI and 14 stable CAD patients with no history of infarction. To further investigate the temporal dynamics of candidate gene expression at single-cell resolution, we integrated the GSE163129 single-cell RNA-Sequencing (scRNA-seq) dataset [17]. This 10× Genomics dataset profiles CD45^+^ immune cells isolated from infarcted C57BL/6 mouse hearts at four time points post-MI (days 1, 3, 5, and 7). This cross-species, single-cell validation approach was employed to confirm the robustness of our findings and to explore how the immune microenvironment evolves during the MI progression.

### 2.2. Identification of Differentially Expressed Genes (DEGs)

Differential expression analysis was performed independently on the GSE60993 and GSE59867 transcriptomic profiles using the ‘limma’ (Linear Models for Microarray Data) R package (R version 3.60.6) [18]. To identify biologically meaningful and statistically robust candidates, DEGs were selected based on a stringent threshold of an absolute fold change exceeding 1.5 (|FC| > 1.5) and *p*-value below 0.05 (*p* < 0.05). The ‘ggplot2’ package (R version 4.0.0) was used to generate volcano plots mapping the global distribution of these genes, while the ‘ComplexHeatmap’ package (R version 2.21.1) [19] was employed to display the top 20 genes, comprising 10 upregulated and 10 downregulated genes ranked by the magnitude of their log2 fold change (|log_2_FC|).

### 2.3. Immune Cell Infiltration Analysis

The ‘CIBERSORT’ algorithm (R version 0.1.0) [20] with the LM22 signature matrix was applied to the GSE59867 and GSE60993 bulk transcriptomic data to estimate the relative proportions of 22 immune cell types. To ensure analytical reliability, only samples with deconvolution *p* < 0.05 and cell types with non-zero estimated proportions were included in downstream analyses. The profiling encompassed a broad spectrum of immune lineages, including B cells, T cells, natural killer (NK) cells, monocytes, macrophages, dendritic cells, mast cells, eosinophils, and neutrophils. Subsequently, the ‘corrplot’ R package (R version 0.92) was used to generate a correlation heatmap, illustrating potential synergistic or antagonistic interactions among immune cell subpopulations. Finally, the ‘ggplot2’ package was used to generate boxplots, visualizing distribution differences in immune cell infiltration across cohorts.

### 2.4. Functional Enrichment Analysis

To elucidate the biological functions of the identified DEGs, Gene Ontology (GO) enrichment analysis was performed using the ‘clusterProfiler’ R package (R version 4.8.3) [21]. Enriched terms were considered statistically significant at an adjusted *p*-value threshold of <0.05. The most significantly enriched functional categories were visualized in bar plots to highlight key molecular pathways and biological processes potentially involved in the progression of AMI.

### 2.5. Machine Learning-Based Feature Gene Selection

To identify a robust and efficient gene signature for AMI diagnosis, three machine learning algorithms were applied: support vector machine-recursive feature elimination (SVM-RFE), random forest (RF), and least absolute shrinkage and selection operator (LASSO). This integrated approach was designed to minimize algorithmic bias and enhance the biological relevance of the selected features. An SVM-RFE model with a linear kernel was constructed using the ‘caret’ package (R version 6.0.94), with feature selection optimized through 5-fold cross-validation (CV). For RF analysis, the ‘randomForest’ package (R version 4.7.1.1) was employed [22] (mtry = 1, ntree = 500), and genes were ranked according to the Mean Decrease Gini index, with the top 10 candidates selected. LASSO regression was performed using the ‘glmnet’ package (R version 4.1.8) [23] with a binomial distribution, and the optimal regularization parameter (λ) was determined via 5-fold CV. The final candidate genes were defined as the intersection of the signatures obtained from all three methods and visualized with a Venn diagram. These overlapping genes were considered definitive candidate genes and used for all subsequent analyses.

### 2.6. Correlation Analysis Between Immune Cells and Candidate Genes

To investigate the relationship between the identified candidate genes and the immune landscape, we integrated transcriptomics signature with CIBERSORT-derived cellular infiltration profiles. Spearman correlation analysis was performed using the ‘corr.test’ function from the ‘psych’ R package (R version 2.4.3) to evaluate pairwise associations between candidate gene expression and immune cell proportions. Corrections were considered statistically significant if the absolute correlation coefficient exceeded 0.3 (|r| > 0.3) and *p* < 0.05. These resulting correlations were visualized using a correlation matrix.

### 2.7. Diagnostic Model Construction and Validation

The clinical utility of the candidate genes was evaluated using both training and validation datasets. Receiver Operating Characteristic (ROC) curves were generated with the ‘pROC’ package (R version 1.19.0.1) to assess the sensitivity and specificity. This analysis was performed on the training cohorts (GSE59867 and GSE60993) and an independent validation cohort (GSE62646), with an area under the curve (AUC) > 0.7 indicating satisfactory predictive performance. To verify transcriptional differences between AMI patients and controls, the Wilcoxon rank-sum test was applied, and *p* < 0.05 was considered statistically significant.

### 2.8. Single-Cell RNA-Sequencing Analysis

The GSE163129 scRNA-seq dataset was analyzed using the ‘Seurat’ R package (R version 5.1.0) [24]. The analysis pipeline included quality control, data normalization, feature selection, dimensionality reduction, and unsupervised clustering. Initial data filtering was applied to retain high-quality transcriptomic profiles and exclude low-quality libraries or potential doublets. Only cells expressing between 200 and 5000 unique genes were kept, and each gene was required to be detected in at least three cells. After normalization, batch effects across datasets were corrected using Reciprocal Principal Component Analysis (RPCA) integration as implemented in Seurat, which identifies mutual nearest neighbors (anchors) between datasets prior to correction. Uniform Manifold Approximation and Projection (UMAP) was then used to visualize the transcriptomic data in a low-dimensional space. Cell clustering was performed using the FindNeighbors and FindClusters functions, and distinct cell subpopulations were identified through manual annotation based on the expression of canonical marker genes.

### 2.9. Nomogram Development

A clinical prediction nomogram was developed using the ‘rms’ R package (R version 6.5.0) based on the candidate biomarkers validated through single-cell analysis. A multivariate logistic regression model was constructed incorporating these biomarkers as independent predictors. Calibration curves were generated to assess the agreement between predicted and observed AMI incidence. To evaluate clinical utility, decision curve analysis (DCA) was performed using the ‘ggDCA’ package (R version 1.2). The net benefit of the model was compared against ‘treat-all’ and ‘treat-none’ strategies, with a higher net benefit indicating that the nomogram offers significant value for clinical decision-making.

### 2.10. Animal Study

Eight-week-old male C57BL/6J mice (23 ± 2 g) were purchased from Guangdong Vital River Laboratory Animal Technology Co., Ltd. (Foshan, China). The animals were housed in separate cages under a 12-h light–dark cycle at 22 ± 2 °C, with free access to food and water.

### 2.11. Animal Model of Myocardial Infarction

Mice were anesthetized with 2% isoflurane during surgical procedures. MI was induced by permanent ligation of the left anterior descending (LAD) coronary artery [25]. During the early recovery phase, mice were placed on a 37 °C heating pad to prevent hypothermia and maintain physiological homeostasis. Sham-operated mice underwent an identical surgical procedure, except that the LAD artery was not ligated.

### 2.12. In Vivo Cardiac Function Assessment by Echocardiography

Transthoracic echocardiography was performed one week post-MI induction. Following removal of thoracic hair, mice were anesthetized with 2% isoflurane and maintained at 1.5% isoflurane under continuous monitoring of respiration and heart rate (maintained between 400–650 bpm). Two-dimensional guided M-mode tracings at the papillary muscle level were acquired from a parasternal short-axis view using a Vevo 3100 imaging system (FUJIFILM VisualSonics Inc., Toronto, ON, Canada) equipped with a 40-MHz transducer. Left ventricular functional parameters were analyzed offline using Vevo Lab software (Version 5.8.2), based on measurements from three consecutive cardiac cycles.

### 2.13. Hematoxylin and Eosin (H&E) Staining

Heart tissues were fixed in 4% paraformaldehyde for 24 h, processed, and embedded in paraffin. Serial sections of 4 μm thickness were cut using a microtome and stained with H&E following a standard protocol [26]. The stained sections were digitally scanned using an automated slide scanner (VS200, Olympus, Muenster, Germany) for subsequent histopathological evaluation.

### 2.14. Quantitative Real-Time PCR

Total RNA was extracted from the myocardial tissues of MI-model and sham-operated mice using TRIzol reagent (Invitrogen, Carlsbad, CA, USA, Cat. No. 15596018CN). Reverse transcription was performed using HiScript III RT SuperMix for qPCR (+gDNA wiper) (Vazyme, Nanjing, China, Cat. No. R323-01) to synthesize cDNA. Quantitative real-time PCR (qRT-PCR) was carried out using Taq Pro Universal SYBR qPCR Master Mix (Vazyme, Cat. No. Q712-02). Relative gene expression was calculated using the 2^−ΔΔCt^ method, with glyceraldehyde-3-phosphate dehydrogenase (GAPDH) serving as the internal reference gene. The forward and reverse primer sequences used are listed in Supplementary Table S1.

### 2.15. Western Blotting

Total protein was extracted from mouse myocardial tissues by homogenization in radioimmunoprecipitation assay lysis buffer (Maclin, Shanghai, China, R874810) supplemented with 1× protease and phosphatase inhibitors (cOmplete™, Roche, Basel, Switzerland, 04693132001; PhosSTOP™, Roche, Mannheim, Germany, 04906837001, respectively). Lysates were incubated at 4 °C for 60 min, then centrifuged at 13,000× *g* for 15 min at 4 °C. The supernatant was collected, and protein concentrations were determined using a bicinchoninic acid protein assay kit (Seven Biotech, Beijing, China, Cat. No. SW201-02). Equal amounts of protein samples were separated by sodium dodecyl sulfate-polyacrylamide gel electrophoresis and transferred onto polyvinylidene difluoride membranes (Bio-Rad, Hercules, CA, USA, 1620177). After blocking the membranes with 5% non-fat milk, membranes were incubated overnight at 4 °C with primary antibodies against MCEMP1 (1:1000, Proteintech, Wuhan, China, 25700-1-AP) and β-tubulin (1:1000, CST, Danvers, MA, USA, 2146S). Following three washes, membranes were incubated with a secondary horseradish peroxidase-conjugated anti-rabbit IgG antibody (1:5000, CST, 7074S) for 1 h at room temperature. Protein signals were visualized using enhanced chemiluminescence detection reagents (Bio-Rad, 1705062) and captured with a ChemiDoc MP Imaging System (Bio-Rad, 12003153). Band intensities were quantified using ImageJ software (Version 1.54f) and normalized to β-tubulin expression.

### 2.16. Statistical Analysis

Statistical analyses were conducted using R (R version 4.4.1) and GraphPad Prism (Version 9.5.0). For bioinformatics analyses, two-group comparisons were performed using the Wilcoxon rank-sum test, and associations were assessed via Spearman’s rank correlation, with correlations considered biologically meaningful at *p* < 0.05 and |r| > 0.3. For experimental data normality was evaluated using the Shapiro–Wilk test, and homogeneity of variances was assessed with the Brown-Forsythe test. Comparisons between two groups were made using unpaired two-tailed Student’s *t*-tests. For multiple-group comparisons, one-way ANOVA was applied when variances were equal, while Welch’s ANOVA followed by Dunnett’s T3 post hoc test was used when variances were unequal. Data are presented as mean ± standard deviation (SD), and *p* < 0.05 was considered statistically significant.

## 3. Results

### 3.1. Integrated Strategy for AMI Biomarker Discovery and Validation

**Figure 1:**
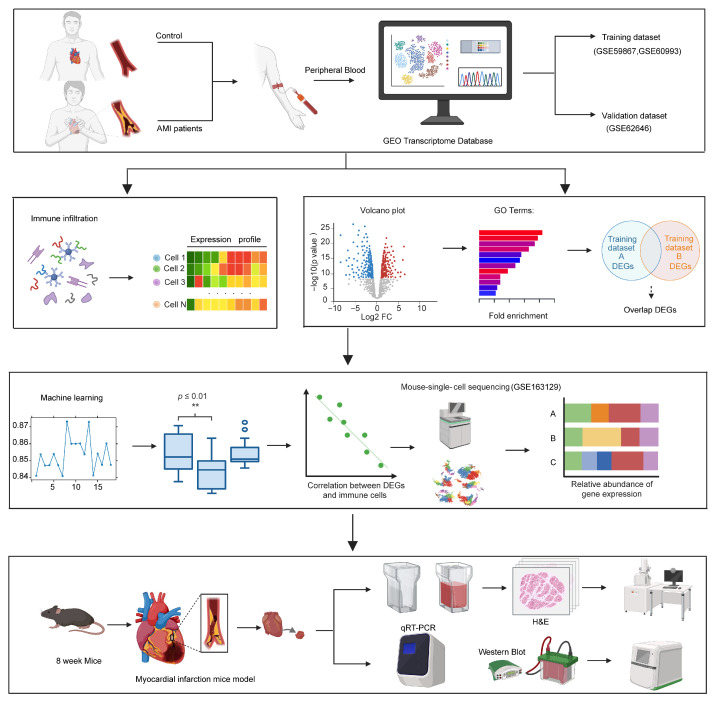
Schematic overview of the multi-stage strategy for the discovery and validation of potential biomarkers in AMI. Peripheral blood transcriptome datasets from patients with AMI were obtained from the GEO database and analyzed through immune infiltration analysis, differential gene expression analysis, GO enrichment analysis, and machine learning-based screening. Candidate genes were further evaluated by correlation analysis with immune cell populations, and validated in mouse single-cell RNA sequencing dataset and animal model of MI.

A multi-omics analysis was conducted to identify and validate novel biomarkers for AMI, as illustrated in Figure 1. Two peripheral blood whole-transcriptome microarray datasets (GSE60993 and GSE59867) for AMI patients were downloaded from the GEO database and subsequently subjected to immune infiltration analysis. DEGs were subsequently identified, and GO enrichment analysis was performed to elucidate their associated biological processes.

Machine learning algorithms were then applied to select key regulatory genes from the identified DEGs. Correlation analysis was subsequently conducted to examine the relationships between these candidate genes and immune cell populations. Changes in gene expression over time post-MI were further explored using the scRNA-seq dataset GSE163129. Finally, a clinical prediction model was developed to assess the diagnostic value of the candidate biomarkers, with their in vivo expression validated in a mouse model of AMI.

### 3.2. Peripheral Immune Profiling Reveals Consistent Neutrophil Enrichment in AMI

**Figure 2:**
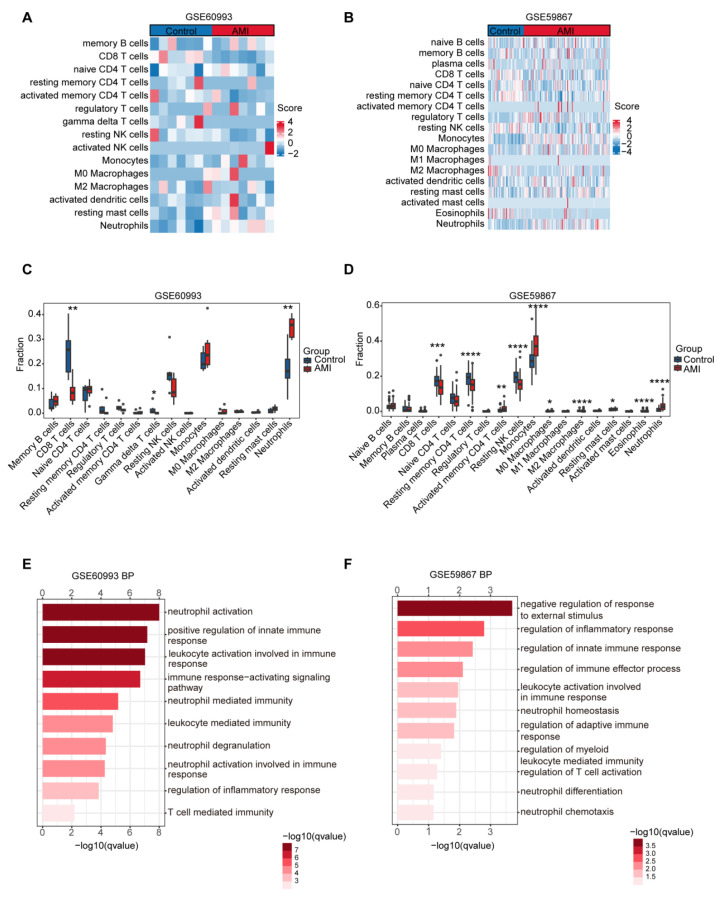
Immune cell infiltration profiles and functional enrichment analysis in AMI. (**A**,**B**) GSE60993 (**A**) and GSE59867 (**B**). Each column corresponds to an individual sample, each row represents a distinct immune cell type, and color intensity indicates the evaluated scores. (**C**,**D**) Box plots comparing the relative proportions of infiltrating immune cells between AMI and control groups in GSE60993 (**C**) and GSE59867 (**D**). (**E**,**F**) GO enrichment analysis of DEGs from GSE60993 (**E**) and GSE59867 datasets (**F**) showing significantly enriched biological processes. The color gradient reflects the significance level (−log_10_ *q*-value). Statistical comparisons between two groups were performed using the Wilcoxon rank-sum test (**C**,**D**). *p* < 0.05 (*), *p* < 0.01 (**), *p* < 0.001 (***), and *p* < 0.0001 (****).

To characterize immune cell infiltration in AMI, the GSE60993 and GSE59867 datasets were utilized as training cohorts for bioinformatics analysis. Immune cell fractions were then quantified using the CIBERSORT algorithm with the LM22 signature matrix to determine the relative proportions of 22 distinct immune cell types. Among these, heatmaps visualization revealed 15 and 18 immune cell subsets with detectable proportions in GSE60993 and GSE59867, respectively (Figure 2A,B). Wilcoxon rank-sum tests identified significantly reduced levels of CD8^+^ T cells and γδ T cells, alongside elevated neutrophils, in AMI patients from the GSE60993 cohort (*p* < 0.01; Figure 2C). Similarly, analysis of the GSE59867 dataset corroborated a broader state of immune imbalance, characterized by decreased resting memory CD4+ T cells, natural killer (NK) cells, M2 macrophages, and eosinophils, accompanied by increased monocytes and neutrophils (*p* < 0.0001; Figure 2D). Notably, both datasets consistently demonstrated a significant elevation in neutrophils, establishing neutrophilia as the dominant immune signature in AMI and a key driver of post-ischemic inflammation.

### 3.3. Transcriptomic Profiling Identifies Neutrophil Activation Pathways in AMI

After data normalization, DEGs between AMI patients and controls were identified using thresholds of |FC| > 1.5 and *p* < 0.05. This analysis revealed 1315 DEGs in GSE60993 (774 upregulated, 541 downregulated) and 89 DEGs in GSE59867 (55 upregulated, 34 downregulated), as illustrated in volcano plots (Figure S1A,B). The top 20 genes, comprising the 10 most upregulated and downregulated, based on log_2_FC, were selected for heatmap visualization (Figure S1C,D).

To explore the potential biological functions of these DEGs, GO enrichment analysis was performed. In the GSE60993 dataset, DEGs were primarily enriched in processes related to neutrophil activation and innate immune signaling (Figure 2E). Consistently, DEGs from the GSE59867 dataset were predominantly associated with immune signaling, systemic inflammatory response, and neutrophil homeostasis (Figure 2F). Together, these cellular and transcriptomic findings indicate that the progression of AMI is closely linked to neutrophil-mediated immune responses.

### 3.4. Identification of Candidate Neutrophil-Related Biomarkers for AMI

**Figure 3:**
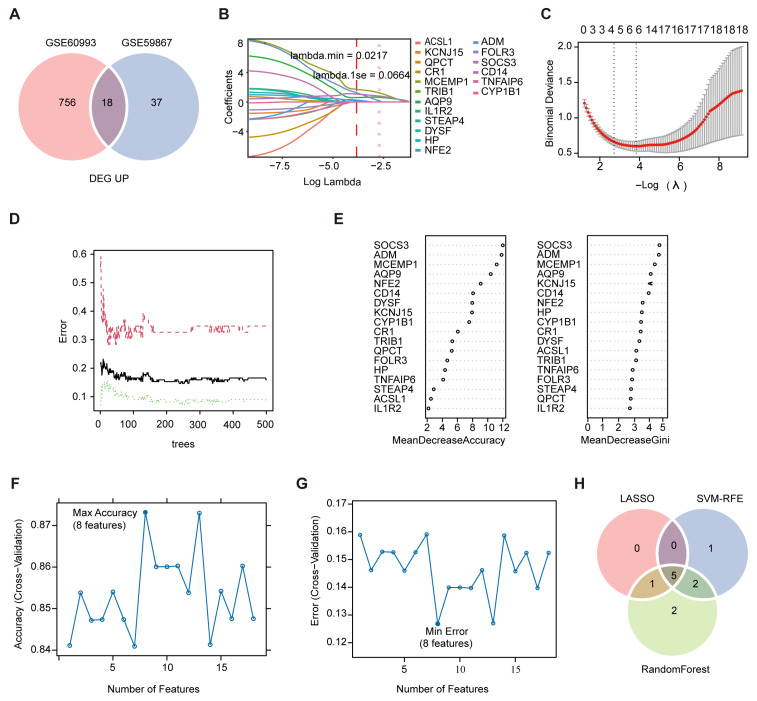
Identification of candidate AMI biomarkers using machine learning algorithms. (**A**) Venn diagram depicting the overlap of upregulated DEGs between the training datasets GSE60993 and GSE59867, identifying 18 commonly dysregulated genes. (**B**) LASSO logistic regression regularization path displaying coefficient trajectories across the log(λ) penalty parameter; vertical dashed lines represent the optimal λ values corresponding to the minimum CV error (λ_.min_ = 0.0217) and the most regularized model within one standard error (λ_.1se_ = 0.0664). (**C**) Five-fold CV mean squared error curve. Numbers along the top indicate the number of genes with non-zero coefficients at each λ, with the red dot marking λ_.min_. (**D**,**E**) Random Forest analysis in the training dataset GSE59867. Out-of-bag (OOB) error rate as a function of the number of decision trees; the black line represents the overall OOB error rate, while the green and red lines represent the OOB error rates for the MI and control classes, respectively (**D**); variable importance metrics for the top genes, ranked by Mean Decrease in Accuracy (Left panel) and Mean Decrease in Gini index (Right panel) (**E**). (**F**,**G**) SVM-RFE analysis in the training dataset GSE59867. Cross-validation accuracy across different subset sizes (**F**) corresponding error rate curve identifying the gene subset with the lowest prediction error (**G**); the solid blue dot indicates the optimal gene set size achieving the highest accuracy (**F**) and lowest prediction error (**G**). (**H**) Venn diagram illustrating the intersection of candidate genes selected by all three machine learning algorithms (LASSO, Random Forest, and SVM-RFE).

To identify robust gene signatures for AMI prediction, we examined the overlap between DEGs from the GSE60993 and GSE59867 datasets. This analysis revealed 18 commonly upregulated (Figure 3A) and 5 commonly downregulated genes (Figure S2). To improve the rigor and robustness of candidate selection, the 18 overlapping upregulated genes were further prioritized by applying three distinct machine learning algorithms to the GSE59867 training dataset. LASSO regression using 5-fold cross-validation selected 6 genes based on the minimum error rate (λ.min = 0.0217; Figure 3B,C). Random forest identified the top 10 genes ranked by importance scores (Figure 3D,E), and SVM-RFE yielded 8 genes with optimal classification accuracy and the lowest error rate (Figure 3F,G). The intersection of the genes selected by all three algorithms yielded five candidate genes, including MCEMP1, AQP9, NFE2, ADM, and SOCS3 (Figure 3H), which were selected as candidate genes for subsequent analyses.

### 3.5. Diagnostic Performance of Candidate Genes and Neutrophil Correlation

**Figure 4:**
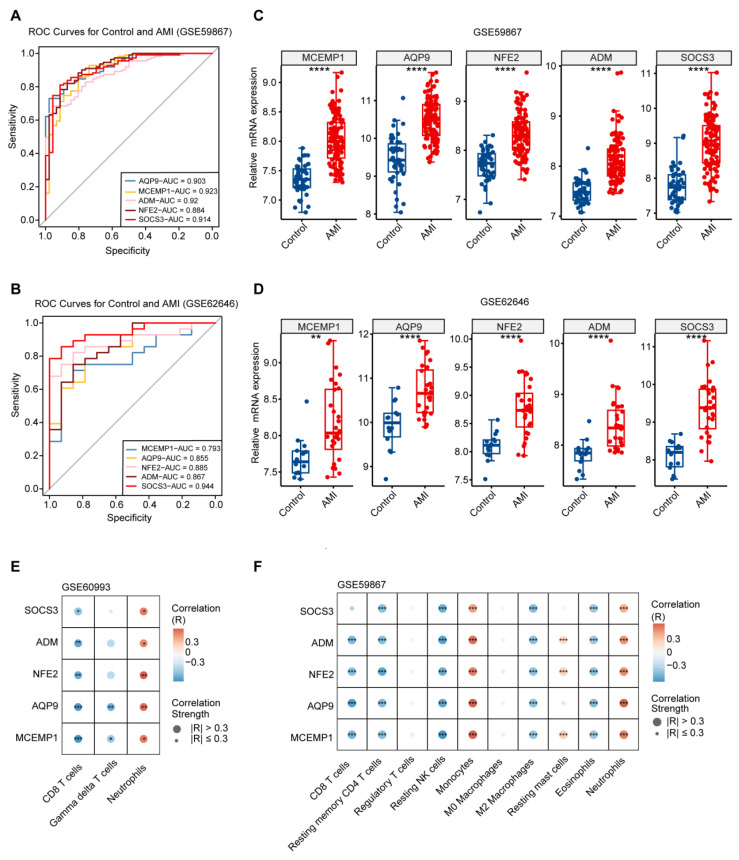
Translational validation of a neutrophil-derived five-gene signature for AMI diagnosis. (**A**,**B**) ROC curves evaluating the diagnostic performance of the five-gene signature in the training dataset GSE59867 (**A**) and the validation dataset GSE62646 (**B**). ROC curve demonstrating diagnostic value. (**C**,**D**) Box plots comparing the expression levels of the five individual genes between AMI patients and controls in GSE59867 (**C**) and GSE62646 (**D**). (**E**,**F**) Correlation analysis between the five candidate genes and the abundance of infiltrating immune cells in GSE60993 (**E**) and GSE59867 datasets (**F**), estimated by CIBERSORT. Dot color represents the Spearman correlation coefficient (R; red: positive correlation, blue: negative correlation). Dot size reflects the magnitude of the correlation (large circles: |r| > 0.3; small circles: |r| ≤ 0.3). Statistical comparisons between two groups were performed using the Wilcoxon rank-sum test; *p* < 0.05 (*), *p* < 0.01 (**), *p* < 0.001 (***), and *p* < 0.0001 (****).

To assess the diagnostic robustness of the five candidate genes for AMI, we calculated the AUC for each gene based on ROC analysis. In the GSE60993 (Figure S3A) and GSE59867 (Figure 4A) training datasets, all five genes exhibited high diagnostic accuracy, with AUC values exceeding 0.8. These findings were further validated in an independent cohort (GSE62646), where each gene maintained strong predictive performance (AUC > 0.7; Figure 4B). Collectively, these results demonstrate that the identified genes possess robust predictive performance for AMI.

The expression levels of the five candidate genes from peripheral blood were further determined in AMI patients and controls. In both the GSE60993 (Figure S3B) and GSE59867 datasets (Figure 4C), all genes were significantly upregulated in the AMI group, a finding consistently validated in the independent GSE62646 dataset (Figure 4D), indicating stable and reproducible upregulation. Moreover, the expression of each gene was strongly and positively correlated with neutrophil infiltration across the two independent datasets (Figure 4E,F), suggesting that this association represents a key biological feature of AMI.

### 3.6. Neutrophils Predominate the Early Inflammatory Phase of Murine MI

**Figure 5:**
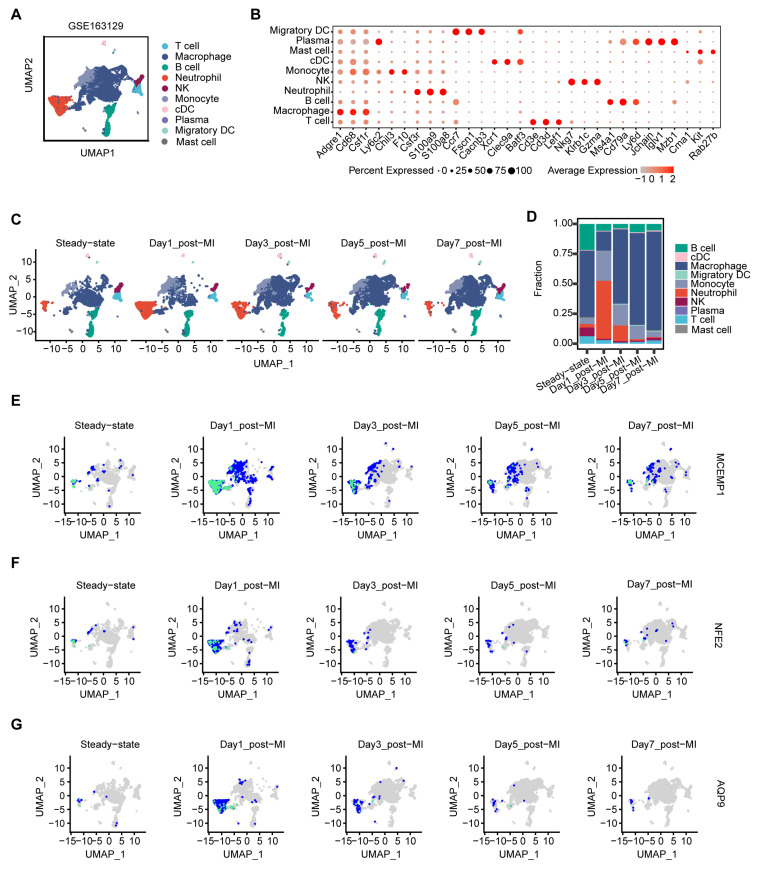
Single-cell transcriptomic profiling of immune cell dynamics in a murine model of MI. (**A**) UMAP visualization of 10 distinct immune cell populations identified in murine cardiac tissue by scRNA-seq. Cell types were annotated based on the expression of canonical marker genes. (**B**) Dot plot showing the expression of canonical marker genes across annotated cell clusters. Dot size represents the percentage of cells expressing each gene within a cluster, while color intensity indicates the mean normalized expression level. (**C**) Temporal distribution of immune cell subsets across post-infarction time points (sham control, days 1, 3, 5, and 7) projected onto the UMAP embedding. (**D**) Stacked bar plot showing the dynamic changes in the relative proportions of immune cell populations over the post-MI time course. (**E**–**G**) Feature plots depicting the spatial expression patterns of the key genes MCEMP1 (**E**), NFE2 (**F**), and AQP9 (**G**) across the UMAP embedding at days 1, 3, 5, and 7 post-MI.

To contextualize peripheral blood findings within the disease microenvironment, we analyzed the GSE163129 scRNA-seq dataset from a mouse model of MI. Following batch integration, 29 cell clusters were identified, with validation confirming uniform cell distribution across batches and no significant batch-driven clustering (Figure S4A,B). Based on these results, we established a baseline immunological landscape of the heart under steady-state conditions. Using canonical cell type-specific marker genes, the 29 clusters were annotated as 10 major cell lineages (Figure 5A). Clusters 5, 6, and 15 exhibited highly expressed neutrophil-specific genes such as S100a8, S100a9, and Csf3r [27,28,29], leading to their classification as the neutrophil population (Figure 5B). The temporal evolution of the cardiac immune microenvironment was then traced across five time points: steady state and days 1, 3, 5, and 7 post-MI (Figure 5C).

Dynamic profiling revealed a rapid influx of neutrophils and monocytes beginning on day 1 post-MI, followed by a gradual decline from day 3 onward. This expansion was accompanied by a sustained relative decrease in T cells, B cells, and NK cells, as well as an initial reduction in macrophages on day 1, which subsequently increased from day 3 (Figure 5C,D). These findings confirm that neutrophils dominate the early inflammatory phase following MI.

### 3.7. Neutrophil-Enriched Candidate Genes Identified by Single-Cell Analysis in AMI

To confirm the cellular localization of the five candidate genes, we mapped their expression patterns onto UMAP derived from scRNA-seq data. Among these, ADM exhibited low abundance in neutrophils across all time points (Figures S4C and S5A). In contrast, consistent with prior studies linking SOCS3 to AMI-associated inflammation [30,31], SOCS3 was upregulated in the MI mouse model but broadly distributed across multiple immune cell populations, rather than being confined to neutrophils (Figures S4D and S5B). Notably, MCEMP1, NFE2, and AQP9 were predominantly enriched in neutrophil clusters on day 1 post-MI. From day 3 onward, MCEMP1 gradually decreased but remained above steady-state levels, whereas NFE2 and AQP9 sharply declined after their initial peak (Figure 5E–G and Figure S5C–E). The predominant enrichment of these three genes in neutrophils highlights their close association with neutrophil activity during the acute inflammatory phase of AMI.

### 3.8. MCEMP1 Dominates the Predictive Utility of the Three-Gene Nomogram for AMI Risk Assessment

**Figure 6:**
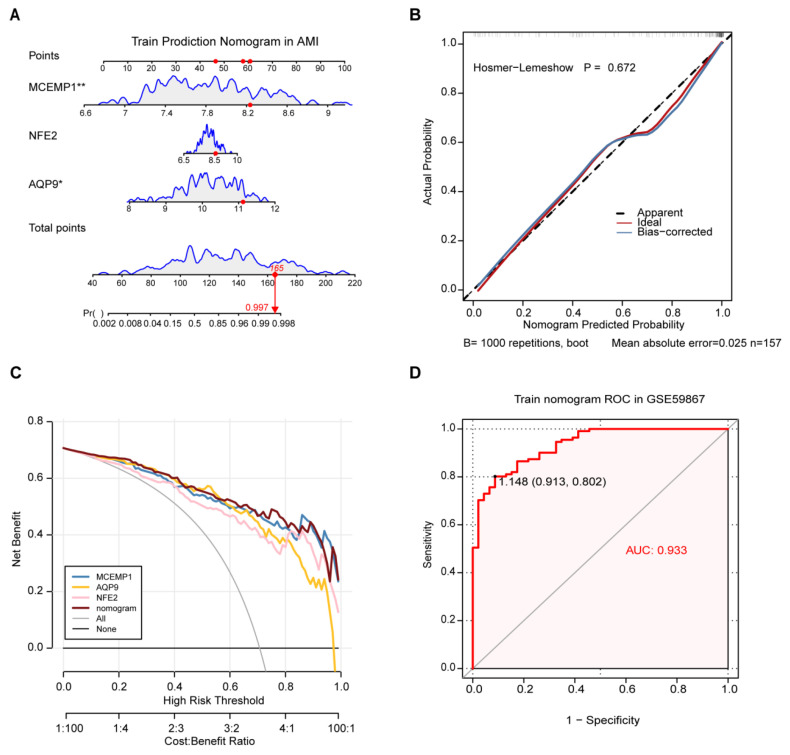
Diagnostic performance and clinical utility of the three-gene signature for AMI. (**A**) Nomogram integrating the expression levels of MCEMP1, NFE2, and AQP9 to predict the probability of AMI. Statistical comparisons for independent predictors were performed using multivariate logistic regression. Thresholds were defined as *p* < 0.05 (*) and *p* < 0.01 (**), with variables at *p* < 0.05 considered statistically significant independent predictors after covariate adjustment. (**B**) Calibration curve assessing the agreement between nomogram-predicted probabilities and actual observed AMI incidence in the training cohort GSE59867. The gray dashed line at 45° represents apparent performance; the blue line shows bias-corrected predictions following 1000 bootstrap resampling steps; the red line indicates ideal prediction. (**C**) Decision curve analysis evaluating the clinical utility of the nomogram. The *y*-axis represents the net benefit, and the *x*-axis represents the threshold probability. The gray line denotes the “treat-all” strategy, the black horizontal axis denotes the “treat-none” strategies, and the colored curves represent the individual genes as well as the combined nomogram. (**D**) ROC curve of the integrated nomogram model, with an AUC of 0.933.

To quantify the predictive value of the target genes for AMI risk, we constructed a nomogram model using the GSE59867 training dataset, which offers a substantially larger sample size, to preliminarily evaluate the three-gene diagnostic panel. Within this integrated model, MCEMP1 emerged as the dominant contributor, outperforming NFE2 and AQP9 (Figure 6A). The model’s robustness was further validated through calibration curve, DCA, and ROC analyses. Calibration curve analysis, based on 1000 bootstrap resamples, demonstrated that predicted probabilities closely aligned with observed outcomes (Hosmer–Lemeshow *p* = 0.672; mean absolute error = 0.025), indicating excellent calibration (Figure 6B).

Similarly, DCA revealed that the single-gene MCEMP1 model delivered net clinical benefit nearly equivalent to that of the full three-gene nomogram and even surpassed it within the high-risk threshold range of 0.8–0.9 (Figure 6C), underscoring MCEMP1 as the major contribution to the combined model. ROC analysis further showed that the three-gene panel achieved strong discriminative power (AUC = 0.933) (Figure 6D). Collectively, these findings indicate that while the three-gene nomogram performs well, MCEMP1 provides the dominant predictive utility and may serve as a parsimonious biomarker for early AMI risk assessment.

### 3.9. MCEMP1 Shows Early Upregulation in the Heart Tissue of the MI Mouse Model

To validate AMI-associated genes identified through bioinformatics analysis, we established a murine MI model via permanent LAD ligation, with sham-operated mice serving as controls. Model fidelity was assessed at days 1, 3, and 7 post-surgery across functional, histological, and molecular dimensions.

**Figure 7:**
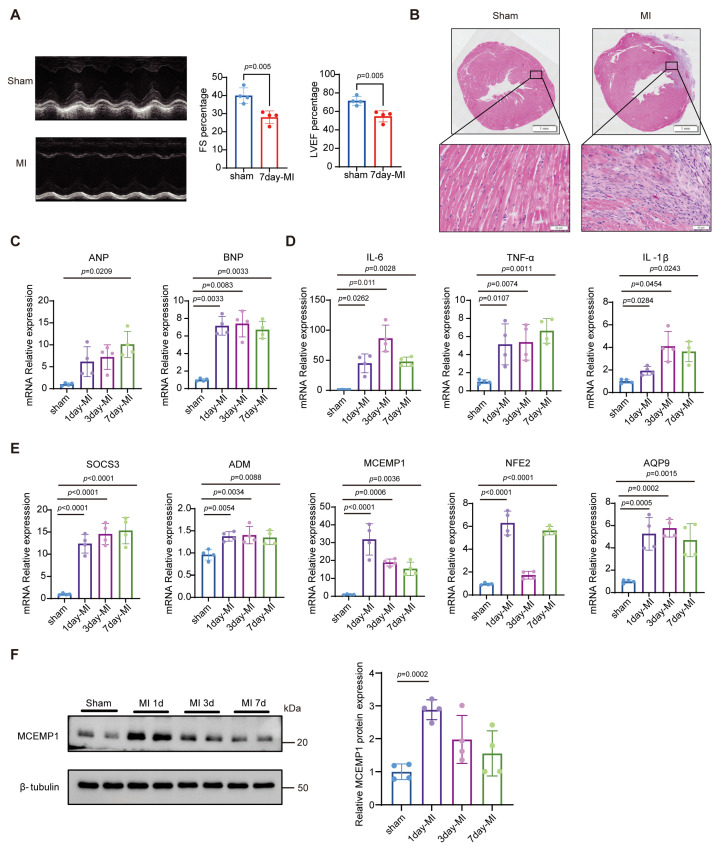
Experimental validation of the murine AMI model and expression profiling of candidate genes. (**A**) Representative M-mode echocardiograms and quantitative analysis of FS and LVEF at 7 days post-infarction (*n* = 4 per group). (**B**) Representative H&E staining of left ventricular tissue from sham and AMI mice at 7 days post-MI (*n* = 4 per group). Scale bar = 50 μm; magnification, 20×. (**C**–**E**) qRT-PCR analysis of mRNA expression levels in left ventricular tissue from sham and MI mice at 1, 3, and 7 days post-surgery (*n* = 4 per group). Myocardial injury markers (**C**) ANP and BNP; inflammatory cytokines (**D**) IL-6, TNF-α, and IL-1β; candidate genes (**E**) SOCS3, ADM, MCEMP1, NFE2, and AQP9. (**F**) Western blotting analysis of MCEMP1 protein expression in myocardial tissue from sham mice and MI mice at 1, 3, and 7 days post-infarction. Band intensities were quantified using ImageJ, normalized to β-tubulin, and expressed as fold change relative to the sham group. Data are presented as mean ± SD. Statistical comparisons were performed using an unpaired two-tailed Student’s *t*-test (**A**) or one-way ANOVA followed by Dunnett’s multiple comparisons test (**C**–**F**).

Functional assessment by echocardiography at day 7 revealed substantially reduced fractional shortening (FS) and left ventricular ejection fraction (LVEF) in MI mice compared with sham controls (Figure 7A), indicating impaired systolic function. Histological examination of H&E-stained cardiac sections further corroborated these findings, showing well-demarcated infarct zones and disrupted myocardial architecture in the MI group (Figure 7B). Together, these multimodal assessments confirm the robust and reproducible establishment of the MI model.

At the molecular level, cardiac injury markers, including atrial natriuretic peptide (ANP) and brain natriuretic peptide (BNP), showed marked upregulation following MI (Figure 7C and Figure S7), consistent with the expected pathophysiological trajectory of post-infarction remodeling. Concurrently, the early inflammatory cascade was rapidly activated, with significant elevation in mRNA levels of the canonical cytokines interleukin-6 (IL-6), tumor necrosis factor-α (TNF-α) and interleukin-1 beta (IL-1β) (Figure 7D).

The expression of the five bioinformatics-prioritized genes was validated at the tissue level. qRT-PCR results showed that SOCS3, ADM, and AQP9 were upregulated from days 1 to 7 post-MI, whereas NFE2 was significantly upregulated only on days 1 and 7. Notably, MCEMP1, which demonstrated high predictive utility in the sequencing data, exhibited a similar expression pattern, increasing on day 1 and gradually decreasing from day 3, though remaining relatively elevated throughout (Figure 7E). MCEMP1 protein expression was further assessed via Western blot analysis, revealing a clear time-dependent upregulation, particularly on day 1 post-MI (Figure 7F and Figure S6). This expression trend validates the bioinformatics-based prediction and reinforces the potential of MCEMP1 as a neutrophil-associated marker in the early phase of MI.

## 4. Discussion

AMI is profoundly shaped by intricate immune regulatory networks, in which neutrophils play a critical role in both the initial inflammatory response and subsequent tissue repair and remodeling [32,33]. Mechanistically, during the early phase of AMI, necrotic cardiomyocytes release damage-associated molecular patterns that engage pattern recognition receptors on resident cardiac macrophages and vascular endothelial cells, thereby activating downstream inflammatory signaling pathways [34]. This cascade promotes the massive release of pro-inflammatory cytokines, granulocyte colony-stimulating factor, and chemokines such as C-X-C motif chemokine ligand 1 (CXCL1), CXCL2, and CXCL8 [35], which collectively promote emergency granulopoiesis in the bone marrow, reduce neutrophil retention in the marrow niche, and enhance neutrophil mobilization into the circulation. These events elicit a rapid surge in peripheral blood neutrophil counts within hours of infarction [36,37,38,39,40,41]. By harnessing potential neutrophil-related signature genes that are altered during early AMI progression, novel biomarkers can be identified for the early diagnosis and prognostic assessment of AMI.

In this study, we initially analyzed publicly available datasets from AMI patients and controls to investigate immune cell abundance during the early phase of AMI. Our findings reveal a significantly higher proportion of neutrophils in the peripheral blood of AMI patients compared to controls, underscoring their pivotal role in AMI development and progression. This observation aligns with previous reports highlighting early neutrophil infiltration in AMI [42,43,44]. Further analysis using three machine learning algorithms identified five candidate genes, MCEMP1, AQP9, NFE2, ADM, and SOCS3, that are closely associated with neutrophil infiltration. The diagnostic performance of these genes was validated through AUC analysis, with all genes demonstrating AUC values greater than 0.7, supporting their potential as reliable biomarkers for AMI.

ScRNA-seq offers unique advantages for screening for disease-related immune biomarkers by enabling transcriptomic profiling of immune cells at single-cell resolution [45]. Meanwhile, leveraging machine learning to identify key molecular features and enhance disease risk prediction accuracy has become a widely adopted approach for biomarker screening [46]. Utilizing bulk transcriptomics analysis and machine learning feature selection, we identified key DEGs in AMI patients. Furthermore, we employed single-cell sequencing to systematically profile the gene expression patterns in neutrophils from a murine model of MI, which confirmed that the expression of all five candidate genes was concurrently upregulated at an early stage post-MI. However, despite prior reports linking ADM and SOCS3 to AMI [30,31,47], our analysis excluded them from further investigation due to low overall expression levels and relatively poor neutrophil specificity, respectively. This refinement narrowed our focus to three key genes, MCEMP1, NFE2, and AQP9, which retain a strong association with neutrophil activities.

Subsequently, we evaluated the diagnostic performance of MCEMP1, NFE2, and AQP9 using a joint prediction model, which revealed that MCEMP1 contributed most to predicting AMI occurrence, followed by AQP9 and NFE2. In the MI mouse model, mRNA expression levels of all three genes were elevated, while MCEMP1 protein expression in the infarcted area was rapidly upregulated on day 1 post-MI and gradually declined on days 3 and 7. This temporal pattern closely parallels the dynamics of neutrophil counts and the extent of inflammatory infiltration, reinforcing the notion that MCEMP1 is tightly linked to early neutrophil-driven inflammation. Therefore, further investigation into the functional role and underlying molecular mechanisms of MCEMP1 in neutrophils is expected to deepen our understanding of inflammatory responses during AMI progression and may provide novel molecular targets for early clinical diagnosis, risk stratification, and preventive strategies.

Among these three genes, NFE2 is a cap’n’collar (CNC) family transcription factor highly expressed in hematopoietic cells and primarily involved in regulating erythropoiesis and oxidative stress responses. It remains highly expressed during CD34^+^ hematopoietic stem cell differentiation into neutrophils [48,49,50], suggesting that it may play a potential role in AMI onset and progression by regulating terminal neutrophil differentiation. Meanwhile, AQP9 is a transmembrane aquaporin that facilitates the transport of water and small solutes and has been implicated in immune-cell polarization and chemotactic migration [51,52]. In fact, AQP9-deficient neutrophils show impaired polarization and reduced migration upon chemokine stimulation [53], supporting its involvement in neutrophil trafficking.

MCEMP1, also a transmembrane protein, is highly expressed in myeloid cells and has been linked to exacerbating vascular inflammation by promoting pro-inflammatory cytokine expression [54]. Although initially characterized in mast cells, where it drives stem cell factor (SCF)-mediated proliferation via extracellular signal-regulated kinase 1/2 (ERK1/2) and protein kinase B (AKT) signaling [55]. Mast cells and neutrophils share key innate immune functions, including rapid degranulation and the release of proteases and cytokines that modulate vascular permeability and inflammation [56]. Notably, during the early phase of AMI, mast cell activation facilitates neutrophil recruitment [57], which in turn amplifies inflammatory signals. Given our observation that MCEMP1 is significantly upregulated in the neutrophils post-AMI and exhibits higher prediction utility, we hypothesize that MCEMP1 may participate in regulating neutrophil activation and migration as well as inflammatory signal amplification during AMI.

Compared with traditional troponin-based approaches, emerging acute coronary syndrome biomarkers, such as growth differentiation factor-15 (GDF-15), soluble suppression of tumorigenicity 2 (sST2), copeptin, and microRNAs, provide valuable mechanistic insights into inflammation, plaque instability, and cardiac stress. Their integration into multi-marker models represents a key direction for improving risk stratification and precision management [58,59,60]. Within this framework, neutrophil-derived biomarkers offer several advantages: they are rapidly released within hours after AMI onset and may rise earlier than cardiac troponins, thereby enabling earlier detection. Furthermore, they can be correlated with disease severity and combined with other clinical parameters to enhance risk stratification and prognostic accuracy [61]. For example, neutrophil gelatinase-associated lipocalin (NGAL), which increases within 2–4 h of AMI onset, has been consistently associated with higher risk of major adverse cardiovascular events, including death, recurrent infarction, and heart failure [61,62], whereas myeloperoxidase (MPO) reflects oxidative and thrombotic activity [58]. In this context, our identified gene MCEMP1, predominantly expressed in neutrophils, may similarly serve as a novel candidate biomarker with potential relevance to early inflammatory activation and prognostic assessment in AMI. Concurrently, anatomical risk scoring tools such as the SYNTAX score have established clinical value in predicting outcomes of coronary artery disease. Integrating our neutrophil-based molecular biomarkers with these existing frameworks may provide additive value, enabling enhanced early risk stratification prior to angiography [63].

Nonetheless, this study has several limitations. No human serum or plasma samples were tested; therefore, the translational relevance of these findings to human AMI remains to be established. Future studies are warranted to evaluate MCEMP1 expression in peripheral blood and to determine its potential as a circulating biomarker. Additionally, the study relies on publicly available datasets with small sample sizes and lacks prospective human cohorts, underscoring the need for future large-scale, independent validation studies to establish MCEMP1, NFE2, and AQP9 as reliable circulating biomarkers for AMI diagnosis. Furthermore, the specific functions and molecular regulatory mechanisms of these genes in neutrophils remain poorly defined. Building on the current data, our future research will focus on systematically elucidating their roles in neutrophil activation, inflammatory regulation, and myocardial injury, aiming to provide a scientific foundation for the development of neutrophil-targeted therapeutic strategies for the treatment of AMI.

## 5. Conclusions

In this study, we employed an integrative multi-omics approach to identify five candidate genes associated with neutrophil infiltration in patients with AMI. Single-cell profiling of infarcted murine hearts revealed that three of these genes (MCEMP1, NFE2, and AQP9) exhibit dynamic expression patterns during the early inflammatory phase following injury. Among them, MCEMP1 demonstrated the strongest correlation with neutrophil activity and contributed most substantially to the diagnostic model. In vivo validation further confirmed marked upregulation of MCEMP1 expression in myocardial tissue after infarction, underscoring its potential as a novel neutrophil-related biomarker for AMI. These findings highlight MCEMP1 as a promising candidate for future investigation in the diagnosis and pathophysiology of AMI.

## Data Availability

All transcriptome datasets used in this study (GSE60993, GSE59867, GSE62646, and GSE163129) were obtained from the publicly accessible GEO database. The code used for data analysis is available from the corresponding author upon reasonable request.

## Supplementary Materials

The following supporting information can be downloaded at: https://www.mdpi.com/article/10.3390/biology15100781/s1, Figure S1: Differential expression analysis of peripheral blood transcriptomes in AMI cohorts; Figure S2: Overlap of downregulated DEGs between AMI cohorts; Figure S3: Diagnostic performance and expression validation of the five-gene signature in the training cohort; Figure S4: ScRNA-Seq quality control and additional gene expression profiling in murine cardiac tissue following MI, Figure S5: Proportion of gene-positive cells per cell type in each group based on scRNA-Seq; Figure S6: MCEMP1 protein expression in independent myocardial tissue samples; Figure S7: Original images of western blots. Table S1: Primer sequences used in the study.
