# Supplementary material for: Uncovering Potential Neutrophil-Related Biomarkers for Early AMI Diagnosis
Source: Biology (Basel). 2026 May 14;15(10):781. doi: 10.3390/biology15100781 (PMC13203664; doi:10.3390/biology15100781)
Supplement: Supplementary file 1 [file biology-15-00781-s001.zip › 20260412 Supplementary Table S1_20260509154857.pdf]

Supplementary Table S1. Primer sequences used in the study.

Primers	Sequence (5'-3')
M-ANP-F	TGGGCTTCTTCCTCGTCTTGG
M-ANP-R	CTCCAGGTGGTCTAGCAGGTTC
M-BNP-F	ATGGATCTCCTGAAGGTGCTGTC
M-BNP-R	AATTGCTCTGGAGACTGGCTAGG
M-TNF-α-F	TCGTAGCAAACCACCAAGTG
M-TNF-α-R	AGATAGCAAATCGGCTGACG
M-IL-1β-F	TGCCACCTTTTGACAGTGATGA
M-IL-1β-R	TGATGTGCTGCTGCGAGATTTG
M-IL-6-F	TAGTCCTTCCTACCCCAATTTCC
M-IL-6-R	TTGGTCCTTAGCCACTCCTTC
M-MCEMP1 -F	CCAACTCAAACTCAGCCAATCAAC
M-MCEMP1 -R	ACGAGAGCAAGGAGAACATACAAC
M-AQP9-F	TGGTGTCTACCATGTTCCTCC
M-AQP9-R	AACCAGAGTTGAGTCCGAGAG
M-NFE2-F	TCCTCAGCAGAACAGGAACAG
M-NFE2-R	GGCTCAAAAGATGTCTCACTTGG
M-ADM-F	CACCCTGATGTTATTGGGTTCA
M-ADM-R	TTAGCGCCCACTTATTCCACT
M-SOCS3-F	ATGGTCACCCACAGCAAGTTT
M-SOCS3-R	TCCAGTAGAATCCGCTCTCCT
