# Supplementary material for: Uncovering Potential Neutrophil-Related Biomarkers for Early AMI Diagnosis
Source: Biology (Basel). 2026 May 14;15(10):781. doi: 10.3390/biology15100781 (PMC13203664; doi:10.3390/biology15100781)
Supplement: Supplementary file 1 [file biology-15-00781-s001.zip › 20260513- Supplementary Figure-QAJ_20260509153533.pdf]

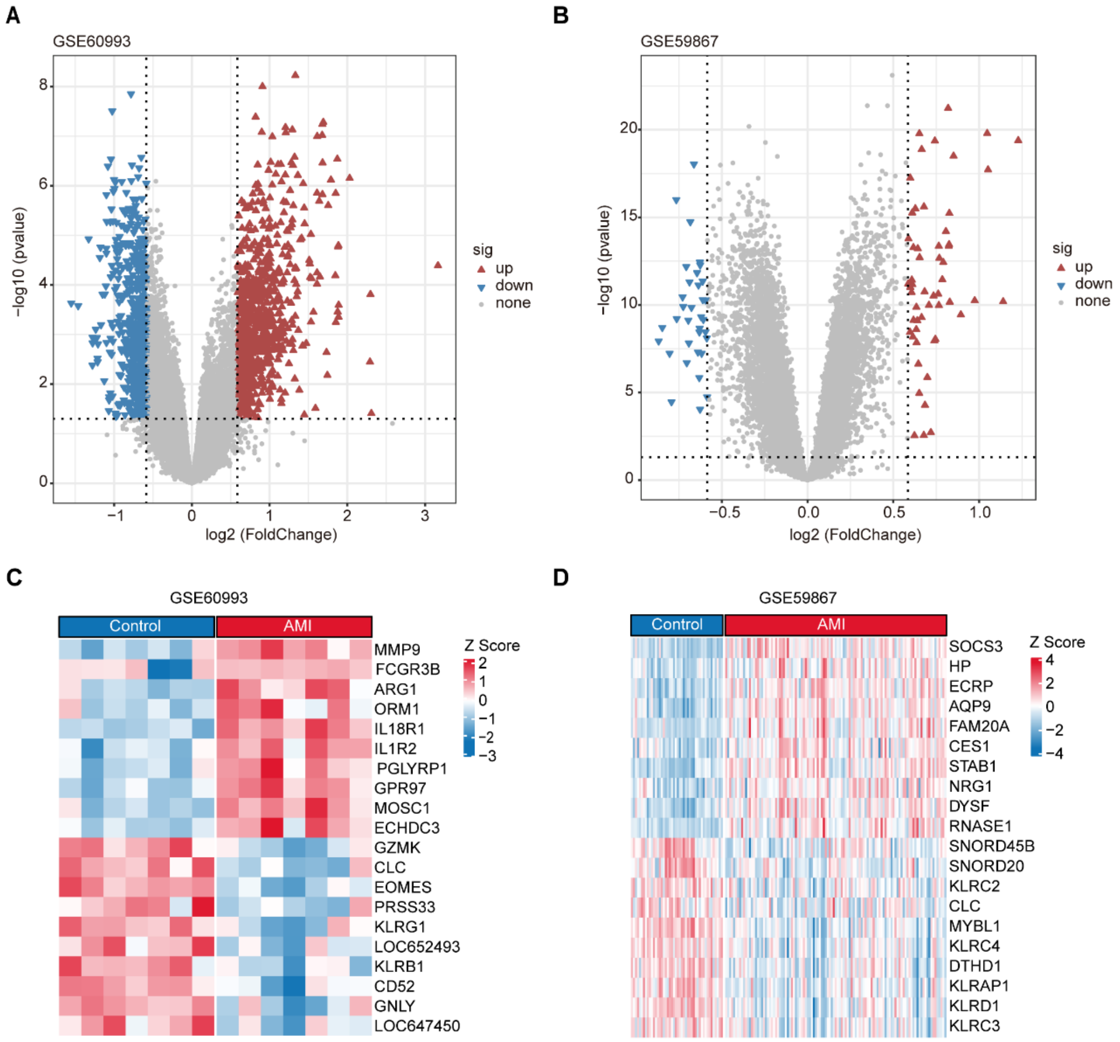

Figure S1. Differential expression analysis of peripheral blood transcriptomes in AMI cohorts.

(A–B) Volcano plots illustrating DEGs in training cohorts GSE60993 **(A)** and GSE59867 **(B)**. Red dots represent upregulated genes ($\log_2 FC > 0.585$, $p < 0.05$); blue dots represent downregulated genes ($\log_2 FC < -0.585$, $p < 0.05$); gray dots indicate non-significant genes. **(C–D)** Heatmaps displaying the expression profiles of the top 10 up and down DEGs ranked by $\log_2 FC$ in GSE60993 dataset **(C)** and GSE59867 dataset **(D)**. Each row corresponds to a single DEGs, and each column represents an individual sample (blue: control; red: AMI). Color intensity reflects the normalized expression level (Z-score), where red denotes high expression and blue denotes low expression.

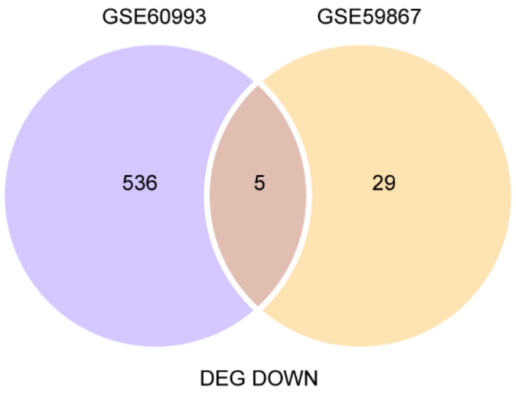

Figure S2. Overlap of downregulated DEGs between AMI cohorts.

Venn diagram depicting the intersection of downregulated genes ($\log_2 FC < -0.585$, $p < 0.05$) identified in the training cohorts GSE60993 and GSE59867. A total of five genes were commonly downregulated across both cohorts.

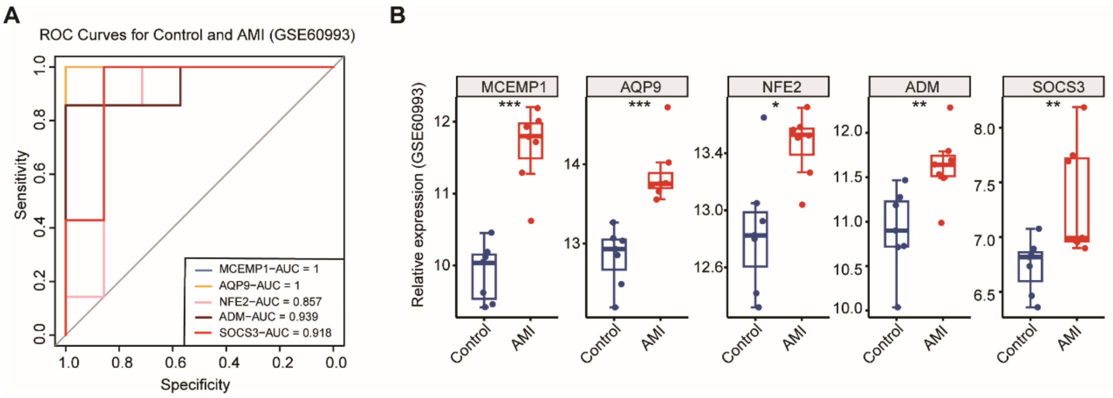

Figure S3. Diagnostic performance and expression validation of the five-gene signature in the training cohort.

(A) ROC curves evaluating the diagnostic accuracy of the five hub genes (MCEMP1, NFE2, AQP9, SOCS3, and ADM) in the training cohort GSE60993. AUC values are indicated in the legend. **(B)** Box plots comparing the expression levels of the five genes between AMI patients and healthy controls in GSE60993 dataset. Data are presented as normalized expression values. Statistical comparisons were performed using the Wilcoxon rank-sum test; $p < 0.05$ (*), $p < 0.01$ (**), $p < 0.001$ (***), and $p < 0.0001$ (****).

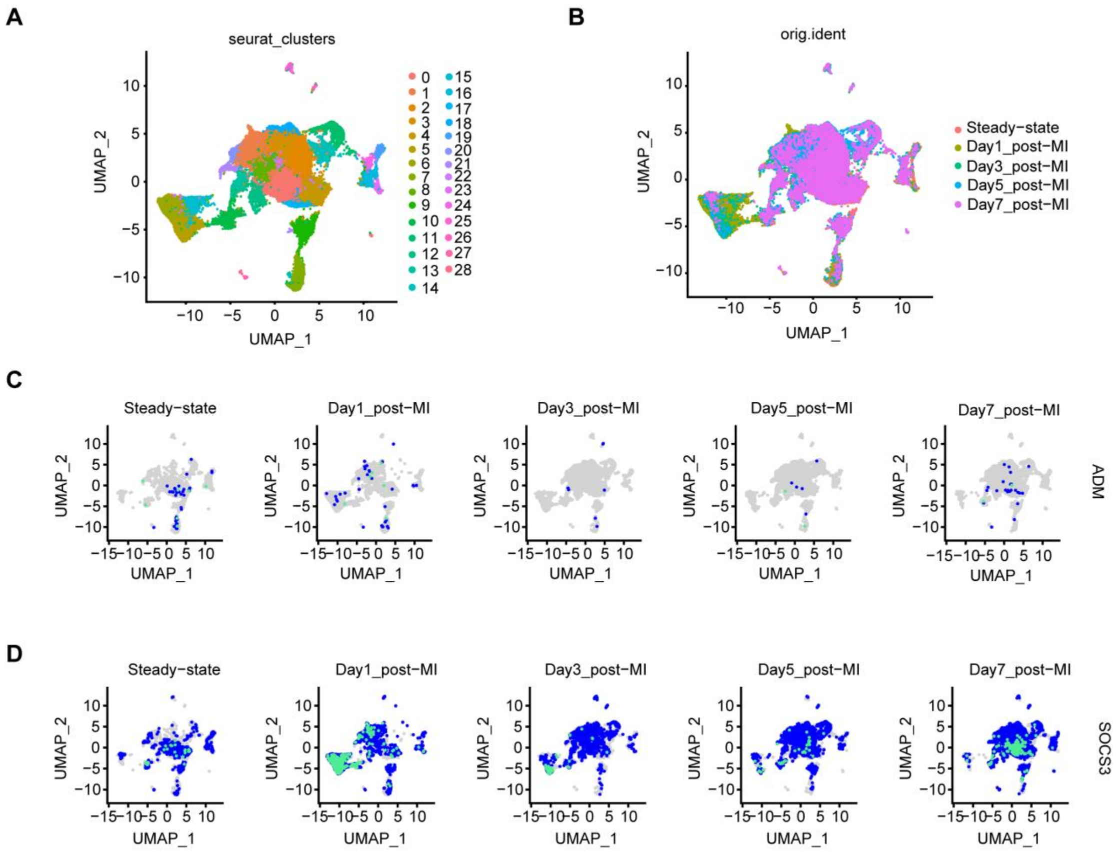

Figure S4. ScRNA-Seq quality control and additional gene expression profiling in murine cardiac tissue following MI.

(A) UMAP visualization of 29 transcriptionally distinct cell clusters in scRNA-seq data from infarcted mouse hearts. Each dot represents a single cell; colors denote cluster identity. **(B)** The UMAP plot demonstrating batch effect correction across individual mouse samples. Each color represents a distinct sample, with dots corresponding to single cells. **(C–D)** Feature plots depicting the spatial expression patterns of ADM **(C)** and SOCS3 **(D)** across the UMAP embedding at days 1, 3, 5, and 7 post-MI.

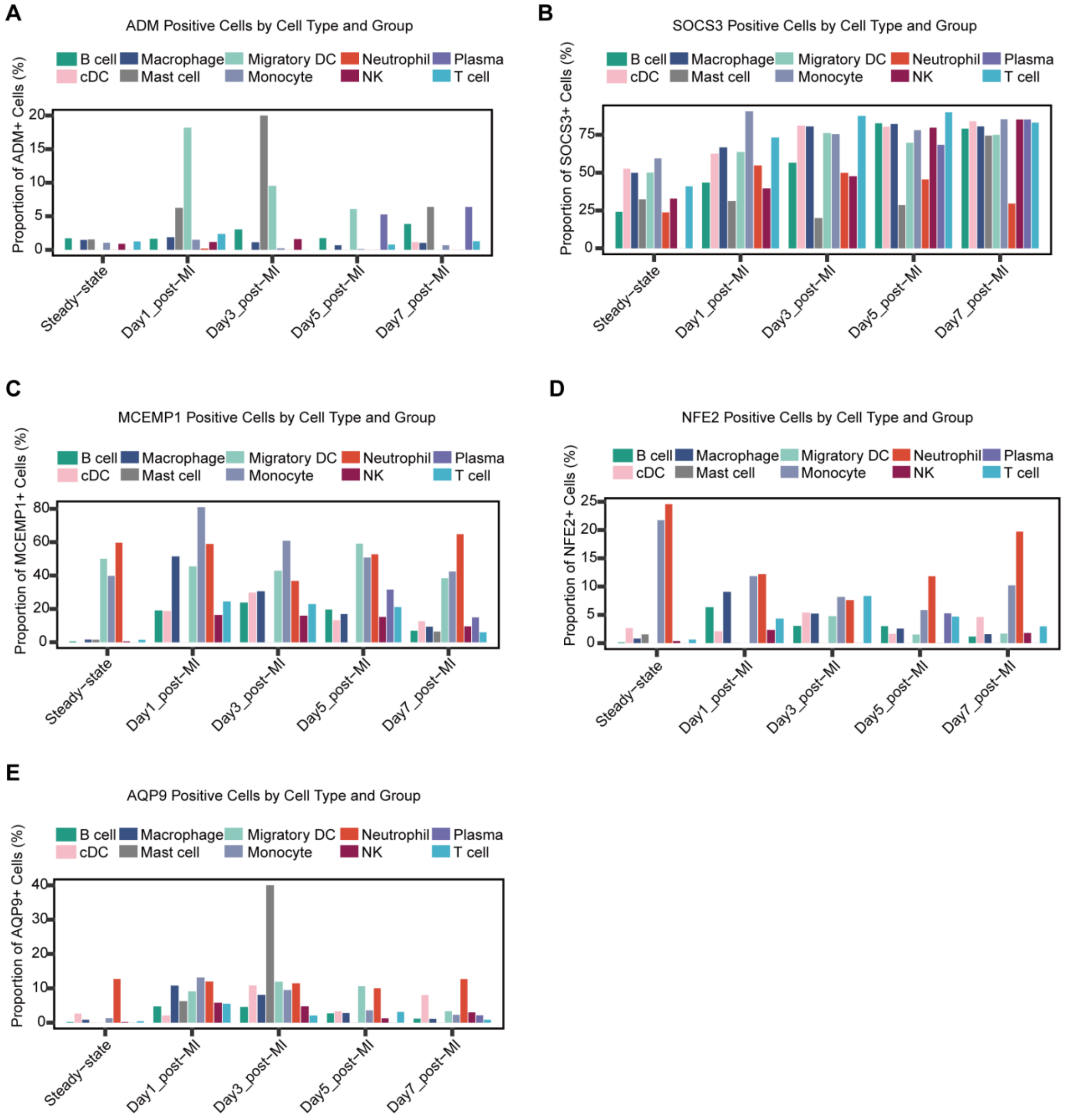

Figure S5. Proportion of gene-positive cells per cell type in each group based on scRNA-Seq.

Bar graphs showing the percentage of cells expressing each target gene (count > 0) within each annotated immune cell population across five time points (steady state, day 1, day 3, day 5, and day 7 post-MI) in the GSE163129 dataset. Panels show the proportion of cells positive for ADM **(A)**, SOCS3 **(B),** MCEMP1 **(C),** NFE2 **(D)**, AQP9 **(E)**, stratified by cell type and time point.

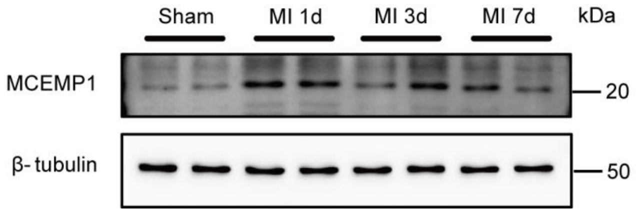

Figure S6. MCEMP1 protein expression in independent myocardial tissue samples. Western blot analysis of MCEMP1 protein expression in left ventricular tissue from sham-operated mice and MI mice at 1, 3, and 7 days post-surgery (n = 4 per group). These samples are independent replicates, distinct from those shown in Figure 7F. Band intensities were quantified using ImageJ, normalized to β-tubulin, and the resulting data were included in the statistical analysis presented in Figure 7F.

A

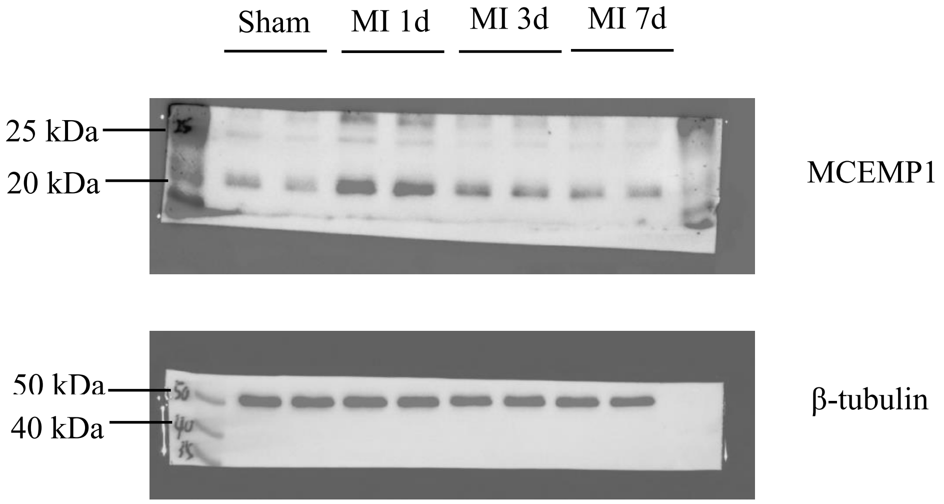

B

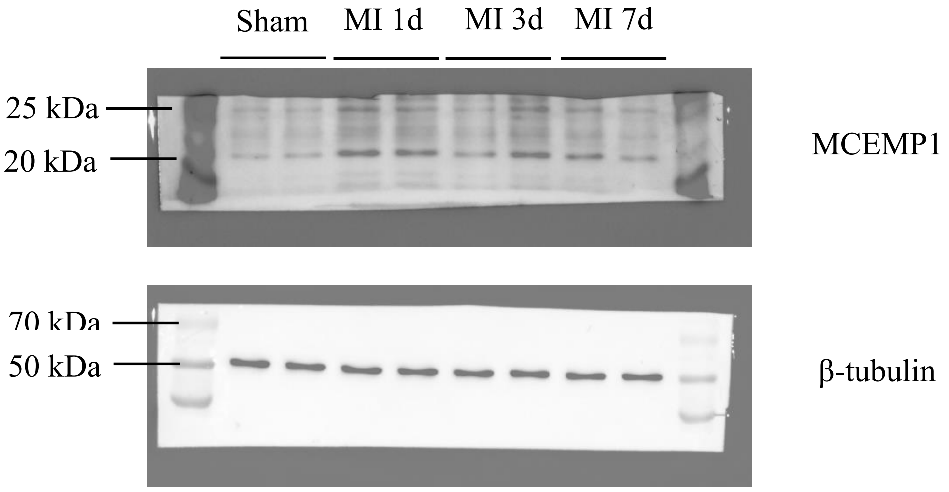

Figure S7. Original images of western blots.
Original western blot images for Figure 7F **(A)** and Figure S6 **(B)** showing MCEMP1 protein expression in left ventricular tissues from sham-operated and MI mice at 1, 3, and 7 days post-surgery (n = 4 per group).
